# Supplementary material for: Type II alveolar cell MHCII improves respiratory viral disease outcomes while exhibiting limited antigen presentation
Source: Nat Commun. 2021 Jun 28;12:3993. doi: 10.1038/s41467-021-23619-6 (PMC8239023; doi:10.1038/s41467-021-23619-6)
Supplement: Supplementary file 1 — Supplementary Information [file 41467_2021_23619_MOESM1_ESM.pdf]

Supplementary Figure 1

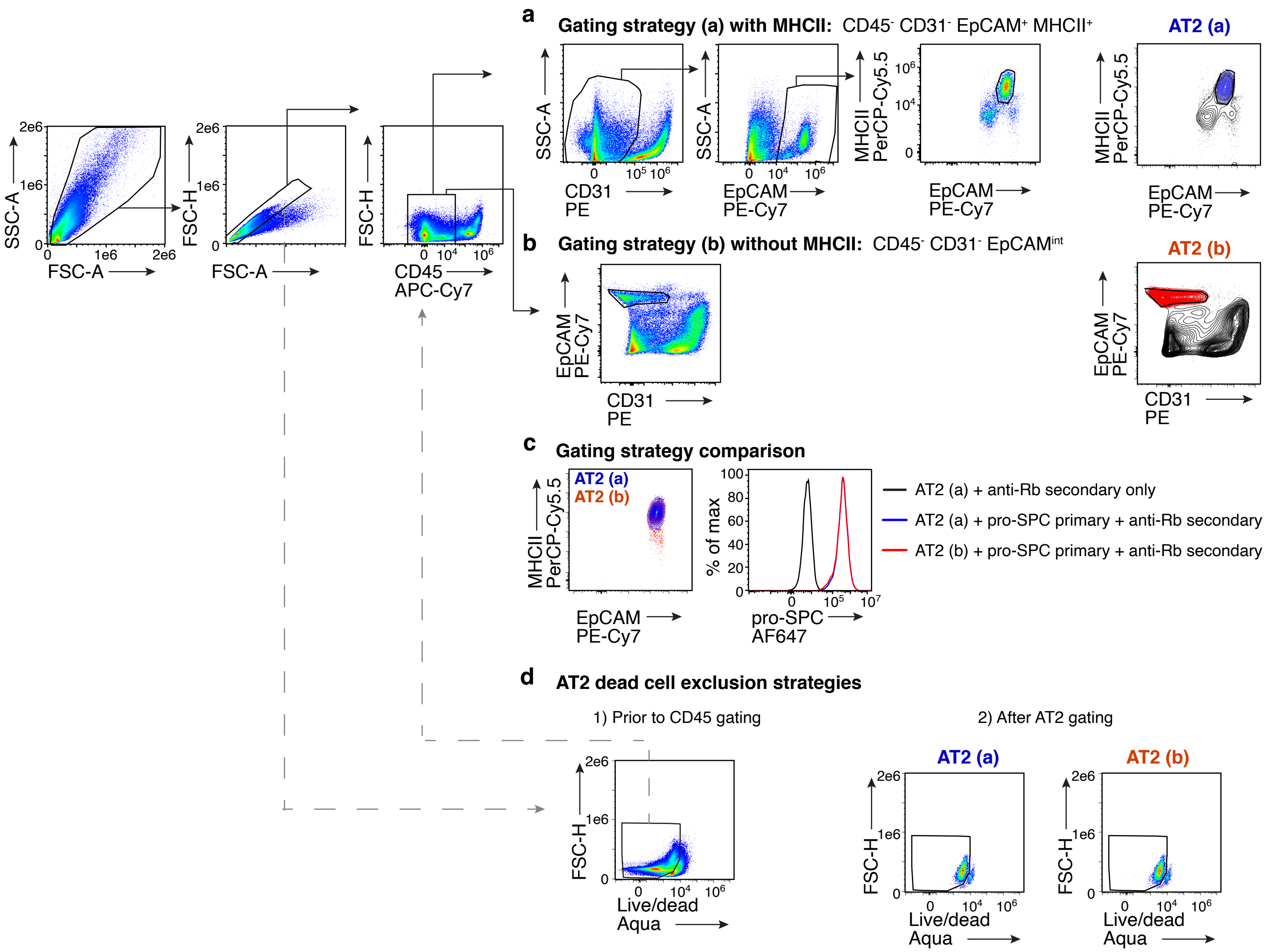

Supplementary Figure 1: Murine AT2s gating strategies.

a,b, Overall gating strategy for flow cytometry and cell sorting of murine AT2s using MHCII as a positive marker (**a**) or without using MHCII as a positive marker (**b**). The right column contour plots are the same as the final pseudocolor plots in the corresponding gating strategies, and they are shown to highlight the final AT2 population defined from each strategy. **c,** Comparison of the two populations of AT2s identified by the two gating strategies (with MHCII [a] in blue, without MHCII [b] in red) in terms of MHCII expression (contour plot, left) or pro-SPC expression (histogram, right). **d**, Example of live/dead gating strategies for AT2 cells used for flow cytometry experiments. Dead cells were excluded in 2 different ways depending on the experiment: 1) after singlets and prior to further gating on $CD45^-$ cells; or 2) as a final gate, as demonstrated for both AT2 cell gating strategies. **a-d**, As detailed in the methods section, for AT2s live/dead staining was used in all flow cytometry analytical experiments but not during cell sorting. In flow cytometry studies where MHCII expression was evaluated (Figures 1a-c,e-g; 3a,c,d; 6d,e, Supplementary Figure 3) and sorting experiments for ELISpot (Figure 5e) and cathepsin assays (Supplementary Figure 5b-c) AT2s were identified using the gating strategy without MHCII as a selection marker as in panel (**b**), as $CD45^-$, $CD31^-$, $EpCAM^{int}$ cells. For all other flow cytometry studies of AT2s, and sorting experiments for DQ-ova assay (Figure 2a), qPCR (Figures 1d, 2c), C57Bl/6 hybridoma assay and Balb/c hybridoma assay (Figures 5b,c), AT2s were identified as $CD45^-$, $CD31^-$, $EpCAM^+$ $MHCII^+$ cells, as in panel (**a**). For organoid culture (Supplementary Figure 8), AT2s were sorted as demonstrated by Paris and colleagues[1] and in Supplementary Figure 12f, as $CD45^-$, $CD31^-$, $Podoplanin^-$, $CD34^-$, $Sca1^-$, $EpCAM^{int}$. **a-d**, These AT2 gating strategies are representative examples. Between experiments, exact cytometer voltages/gains were subject to variation, and the precise fluorophores used for each marker sometimes differed from those displayed here depending on the other markers and fluorophores used within the same experiment. For a full list of antibody-fluorophore conjugates, see Supplementary Table 6.

Supplementary Figure 2

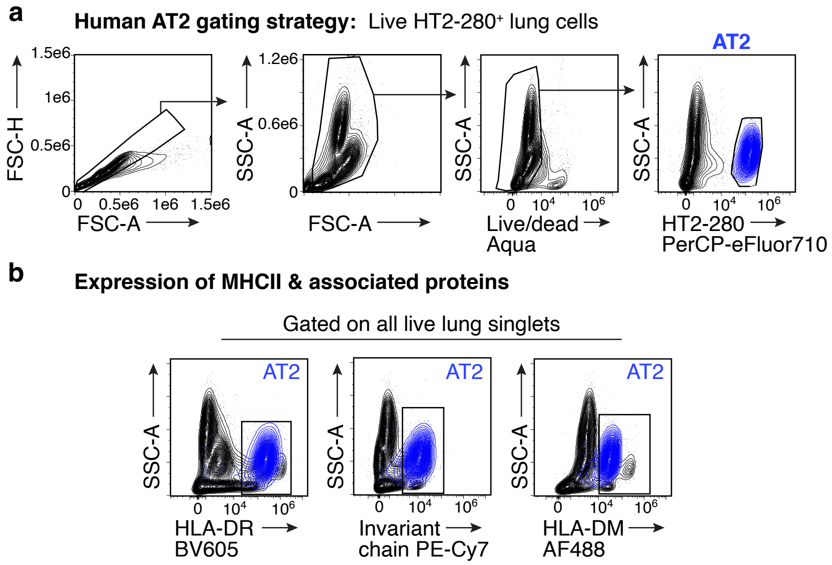

Supplementary Figure 2: Human AT2s express MHCII and associated classical MHCII presentation mediators.

a, Gating strategy for human AT2s. The right-most contour plot highlights the final AT2 population defined by HT2-280^{+} staining. **b,** HLA-DR (left), invariant chain (middle), and HLA-DM (right), protein expression by healthy human distal lung cells (black), quantified by *ex vivo* flow cytometry analysis. The AT2 population, as defined in (**a**), is highlighted in blue. Gates were drawn based on fluorescence minus one (FMO) controls. All plots represent n=1 human donor.

Supplementary Figure 3

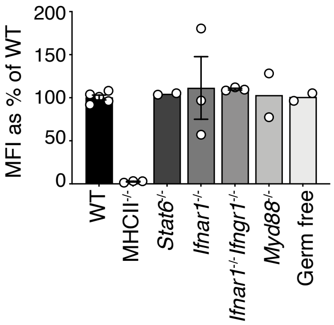

Supplementary Figure 3: AT2s constitutively express MHCII independent of a variety of inflammatory mediators.

MHCII protein expression by AT2s, from naïve B6 WT, MHCII$^{-/-}$ *Stat6*$^{-/-}$, *Ifnar1*$^{-/-}$, *Ifnar1*$^{-/-}$*Ifngr1*$^{-/-}$, *Myd88*$^{-/-}$, and germ-free mice, measured by *ex vivo* flow cytometry analysis. Each symbol reflects MHCII expression on the cell type indicated in n=1 mouse, with n=5 B6 WT mice displayed total pooled from 3 experiments, n=3 MHCII$^{-/-}$ mice displayed total pooled from 3 experiments, n=3 each of *Ifnar1*$^{-/-}$ and *Ifnar1*$^{-/-}$*Ifngr1*$^{-/-}$ mice displayed total pooled from 2 experiments, n=2 each of *Stat6*$^{-/-}$, *Myd88*$^{-/-}$, and germ-free mice displayed from 1 experiment. MHCII expression is quantified as a percentage reflecting the average median fluorescence intensity (MFI) of MHCII on each cell type in a given knockout mouse relative to the MHCII MFI on the same cell type in WT mice. Data are shown as mean plus standard error of the mean (SEM). WT mice are in black, other knockout strains in shades of grey as labeled. Source data are provided as a Source Data file.

Supplementary Figure 4

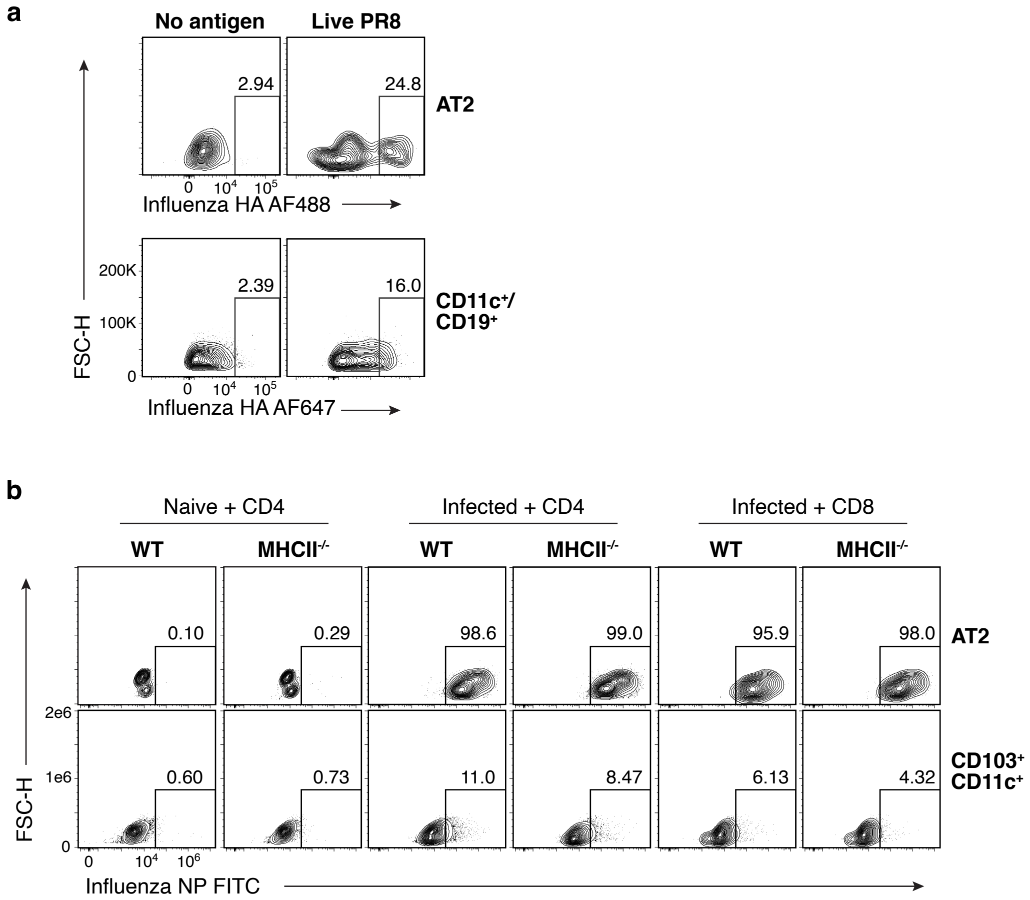

Supplementary Figure 4: AT2s are infected robustly with influenza *in vitro* and *in vivo*.
a, Surface influenza hemagglutinin (HA) expression by B6 AT2s (top) and a mixed population of CD11c^{+} and CD19^{+} lung cells (bottom) sorted from naïve mice then incubated with no antigen (left) or live virus (right) *in vitro* for 14h. These plots reflect the antigen presenting cells used in the hybridoma antigen presentation assay illustrated in Figure 5b. The frequency of HA^{+} cells is shown above the gates, which were drawn based on the no antigen conditions. Two different fluorophore conjugated versions of the same anti-HA antibody were used because the AT2 and CD11c^{+}/CD19^{+} populations were already labeled with different fluorophores (APC and FITC, respectively) from the cell sorting process. **b,** Intracellular influenza nucleoprotein (NP) expression by B6 AT2s (top) and CD103^{+}CD11c^{+} lung cells (bottom) sorted from naïve or flu-infected WT or MHCII$^{-/-}$ B6 mouse lungs 4 dpi and incubated with CD4^{+} and CD8^{+} T cells *in vitro* for 14h (as indicated). These plots reflect the antigen presenting cells used in the ELISpot presentation assay illustrated in Figure 5e. The frequency of NP^{+} cells is shown above the gates, which were drawn based on the naïve conditions.

Supplementary Figure 5

a

Gene	Description	AT2	Alv Mac	CD103⁺ DC	Club Cell
Cd74	Invariant chain	8287	1910	63312	144
Ciita	CIITA	13	2	109	0
H2-Aa	MHCII (H2-A) alpha chain	1528	317	15749	36
H2-Ab1	MHCII (H2-A) beta chain	1648	462	30639	39
H2-DMa	H2-DM alpha chain	126	789	2269	14
H2-DMb1	H2-DM beta 1 chain	137	222	1782	26
H2-DMb2	H2-DM beta 2 chain	10	42	394	5
H2-Oa	H2-O alpha chain	0	4	185	1
H2-Ob	H2-O beta chain	0	0	246	2
Ctsa	Cathepsin A	85	1175	18	116
Ctsb	Cathepsin B	430	1881	459	347
Ctsc	Cathepsin C	1367	1542	291	31
Ctsd	Cathepsin D	198	8487	24	786
Ctse	Cathepsin E	0	0	7	0
Ctsf	Cathepsin F	10	42	0	10
Ctsg	Cathepsin G	0	0	0	0
Ctsh	Cathepsin H	1915	760	486	691
Ctsj	Cathepsin J	0	0	0	0
Ctsk	Cathepsin K	1	591	1	192
Ctsl	Cathepsin L	87	363	6	77
Ctsm	Cathepsin M	0	0	0	0
Ctso	Cathepsin O	12	35	20	22
Ctsq	Cathepsin Q	0	0	0	0
Ctsr	Cathepsin R	0	0	0	0
Ctss	Cathepsin S	7	5435	907	3
Ctsw	Cathepsin W	0	0	0	1
Ctsz	Cathepsin Z	261	1498	764	67
Ifi30	GILT	91	646	583	62
Lgmn	AEP	45	152	39	51

b

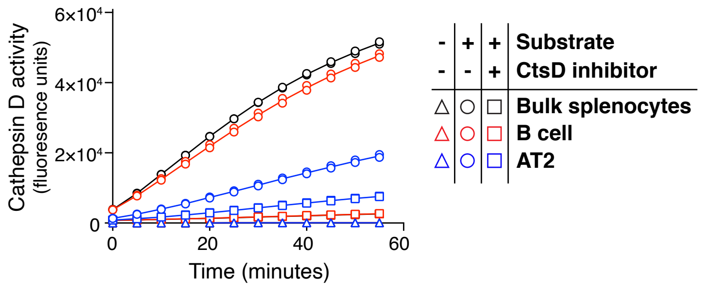

c

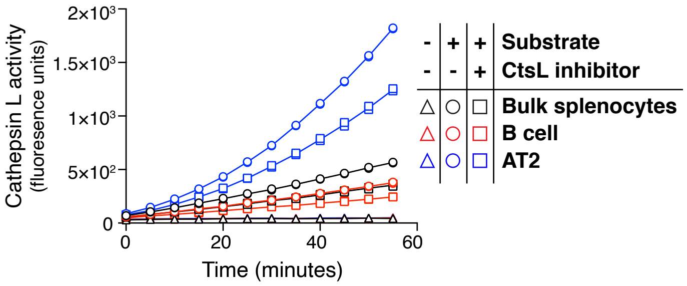

d

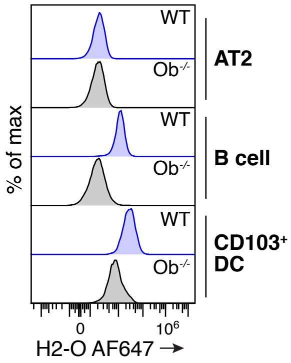

e

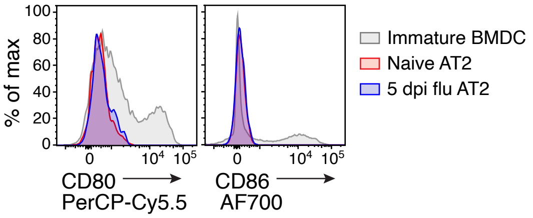

f

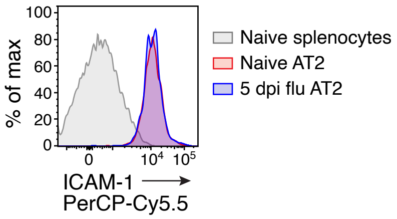

Supplementary Figure 5: AT2s express conventional mediators of MHCII antigen presentation and the costimulatory molecule ICAM-1, but do not express CD80, CD86, or the H2-M antagonist H2-O. **a,** Expression of MHCII-associated genes and proteases in naïve B6 WT AT2s, alveolar macrophages, $CD103^+$ DCs, and club cells, evaluated by RNA-sequencing analysis of data from Ma *et. al.*[2] Expression values are reported as transcripts per million (TPM) and represent the average of n=4 biological replicates for each cell type. **b,** Cathepsin D (CtsD) activity of AT2s (blue), bulk splenocytes (black), and splenic B cells (red) from naïve B6 mice measured by cleavage of a fluorogenic CtsD substrate in the presence (squares) or absence (circles) of the CtsD inhibitor pepstatin A (10 nM), or with no substrate (triangles); symbols shown are 2 technical replicates from n=1 biological replicate, which is representative of n=2 biological replicates for AT2s and n=3 for bulk splenocytes and B cells. **c,** Cathepsin L (CtsL) activity of AT2s (blue), bulk splenocytes (black), and lung B cells (red) isolated from naïve B6 mice, measured by cleavage of a fluorogenic CtsL substrate in the presence (squares) or absence (circles) of a CtsL inhibitor (100 nM), or with no substrate (triangles); symbols shown are 2 technical replicates from 1 biological replicate, which is representative of n=2 biological replicates for splenocytes, n=4 for B cells, and n=1 for AT2s. **d,** Intracellular H2-O protein expression by AT2s, $CD103^+$ DCs, and B cells, from B6 WT (blue) or H2-Oβ deficient ($O\beta^{-/-}$, black) mouse lungs measured *ex vivo* by flow cytometry; histograms represent n=3 WT mice and n=2 $O\beta^{-/-}$ mice from 1 experiment. **e,** Surface expression of CD80 (left) and CD86 (right) by AT2s from naïve mice (red) or mice 5 days post influenza virus infection (blue), and B6 WT BMDCs cultured *in vitro* (grey). **f,** Surface expression of ICAM-1 by AT2s from naïve mice (red) or mice 5 days post influenza virus infection (blue), and B6 WT splenocytes from naïve mice (grey). Histograms represent n=2 mice (naïve) or n=6 mice (flu infected) in total across 2 experiments. Source data are provided as a Source Data file.

Supplementary Figure 6

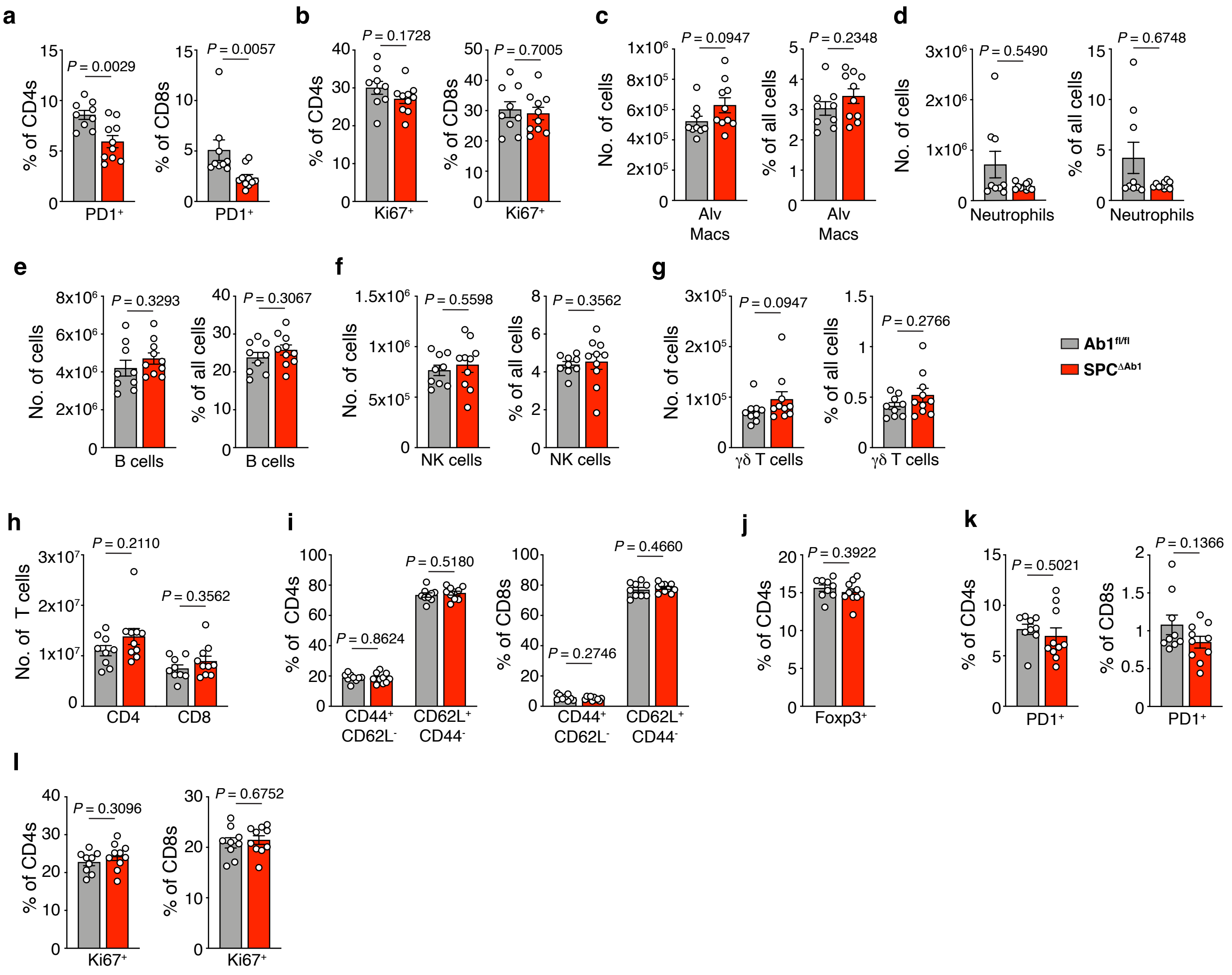

Supplementary Figure 6: Mice lacking AT2 MHCII demonstrate normal immune homeostasis in the lung and spleen.

Ex vivo flow cytometry analysis of naïve 12 week old Ab1$^{fl/fl}$ and SPC$^{\Delta Ab1}$ mice lungs and spleens. **a-b,** Frequency of lung CD4^{+} and CD8^{+} T cells expressing PD1 (**a**), and Ki67 (**b**) **c-g,** Absolute numbers and frequencies of lung alveolar macrophages (**c**), neutrophils (**d**), B cells (**e**), NK cells (**f**), γδT cells (**g**). **h-l,** Absolute number of splenic CD4^{+} and CD8^{+} T cells (**h**), and frequency of splenic CD4^{+} and CD8^{+} T cells expressing CD44, CD62L (**i**), Foxp3 (**j**), PD1 (**k**), and Ki67 (**l**). **a-l**, Each symbol represents n=1 mouse, with n=9 Ab1$^{fl/fl}$ and n=10 SPC$^{\Delta Ab1}$ mice total displayed from 1 experiment, which is representative of 2 similar experiments. Data shown are mean plus SEM, analyzed by unpaired two-tailed Student's *t*-test (**a** [CD4s], **b, c** [%], **e**, **f** [#], **i-l**) or two-tailed Mann-Whitney test (**a** [CD8s], **c** [#], **d**, **f** [%]**, g, h**). Full statistical test results are in Supplementary Table 1. For all panels, Ab1$^{fl/fl}$ are in grey, SPC$^{\Delta Ab1}$ in red. Source data are provided as a Source Data file.

Supplementary Figure 7

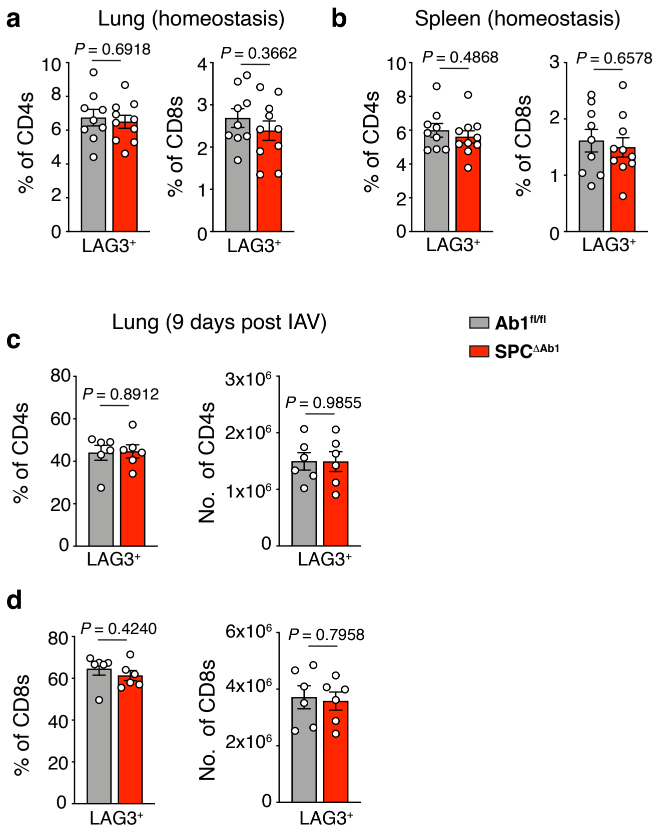

Supplementary Figure 7: Loss of AT2 MHCII does not alter frequencies of LAG3^{+} T cells at homeostasis or after influenza virus infection.

Ex vivo flow cytometry analysis of LAG3 expression by CD4^{+} and CD8^{+} T cells in Ab1$^{fl/fl}$ and SPC$^{\Delta Ab1}$ mice. **a,b,** Frequency of lung (**a**) and spleen (**b**) LAG3^{+} CD4^{+} and CD8^{+} T cells in naïve 12 week old mice; each symbol represents n=1 mouse, with n=9 Ab1$^{fl/fl}$ and n=10 SPC$^{\Delta Ab1}$ mice total displayed from 1 experiment, which is representative of 2 similar experiments. **c,d,** Frequency and number of lung LAG3^{+} CD4^{+} (**c**) and CD8^{+} (**d**) T cells in 8-10 week old mice 9 days post IAV infection; n=6 mice per strain from 1 experiment. **a-d**, Data shown are mean plus SEM, and were analyzed by unpaired two-tailed Student's *t*-test. Full statistical test results are in Supplementary Table 2. For all panels, Ab1$^{fl/fl}$ are in grey, SPC$^{\Delta Ab1}$ in red. Source data are provided as a Source Data file.

Supplementary Figure 8

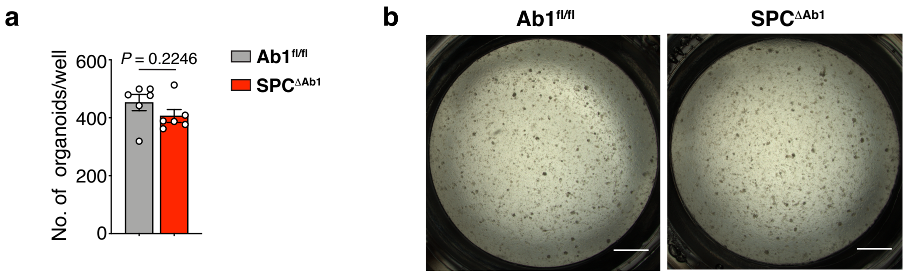

Supplementary Figure 8: Loss of MHCII from AT2s does not inhibit their regenerative capacity. a, b, Numbers (**a**) and representative images (**b**) of lung organoid cultures formed by AT2s sorted from either or Ab1$^{fl/fl}$ and SPC$^{\Delta Ab1}$ mice. Data represent n=6 organoid cultures per strain, grown from AT2s originally sorted from n=1 mouse per strain. **a,** Bars represent mean plus SEM, and the data were analyzed by unpaired two-tailed Student's *t*-test (t=1.294, df=10). Ab1$^{fl/fl}$ are in grey, SPC$^{\Delta Ab1}$ in red. **b,** Scale bar depicts 1000 μm. Source data are provided as a Source Data file.

Supplementary Figure 9

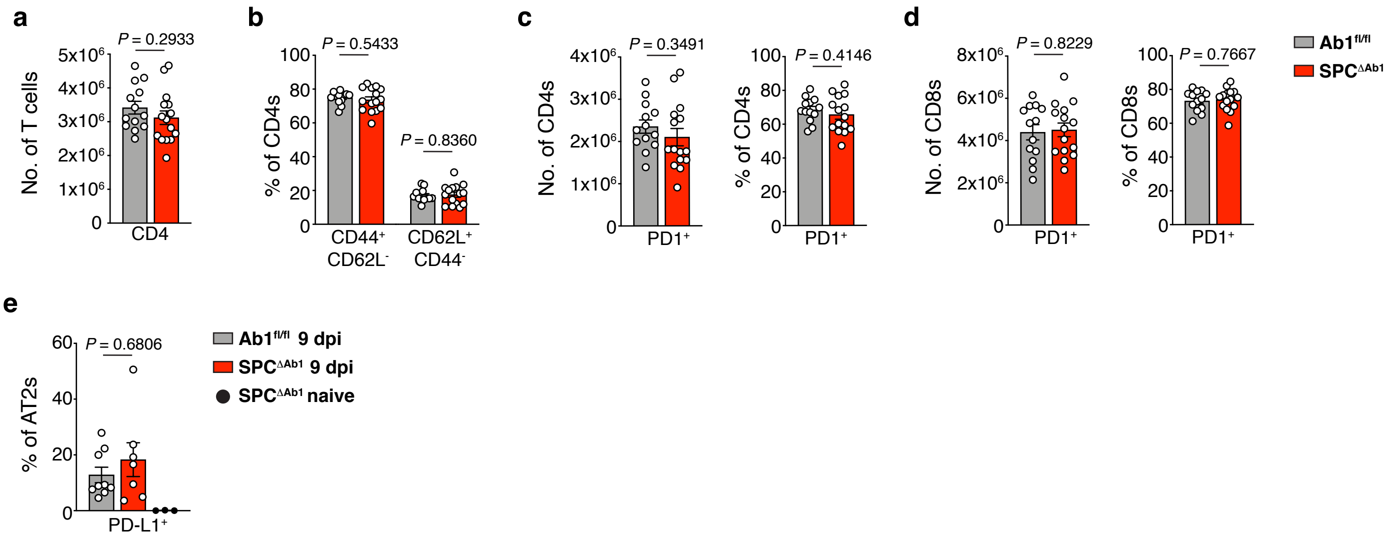

Supplementary Figure 9: Loss of AT2 MHCII does not alter lung bulk or CD44/CD62L-expressing CD4⁺ T cells, PD1-expressing T cells, or PD-L1⁺ AT2s after influenza virus infection.
Ex vivo flow cytometry analysis of PD-L1 expression by AT2s, and CD44, CD62L, and PD1 expression by lung $CD4^+$ and $CD8^+$ T cells. **a-d,** Number of bulk lung CD4+ T cells (**a**), frequency of $CD4^+$ T cells expressing CD44, CD62L (**b**), and number and frequency of lung $PD1^+$ $CD4^+$ (**c**) and $CD8^+$ (**d**) T cells in $Ab1^{fl/fl}$ and $SPC^{\Delta Ab1}$ mice 9 days post IAV infection; each symbol represents n=1 mouse, with n=13 $Ab1^{fl/fl}$ and n=15 $SPC^{\Delta Ab1}$ mice total displayed pooled from 2 experiments. **e,** Frequency of PD-L1⁺ AT2s in $Ab1^{fl/fl}$ and $SPC^{\Delta Ab1}$ mice 9 days post IAV infection and naïve $SPC^{\Delta Ab1}$ mice; each symbol represents n=1 mouse, with n=9 $Ab1^{fl/fl}$ post-IAV, n=7 $SPC^{\Delta Ab1}$ post-IAV, and n=3 $SPC^{\Delta Ab1}$ naïve mice displayed in total pooled from 2 experiments. **a-e**, Data shown are mean plus SEM, and were analyzed by unpaired two-tailed Student's *t*-test (**a, b**[$CD44^-CD62L^+$]**, c, d**), two-tailed Welch's *t*-test (**b** [$CD44^+CD62L^-$]), and two-tailed Mann-Whitney test (**e**). Full statistical test results are in Supplementary Table 5. For panels **a-e**, 9 dpi $Ab1^{fl/fl}$ are in grey, and 9 dpi $SPC^{\Delta Ab1}$ in red. In panel **e**, naïve $SPC^{\Delta Ab1}$ are in black. Source data are provided as a Source Data file.

Supplementary Figure 10

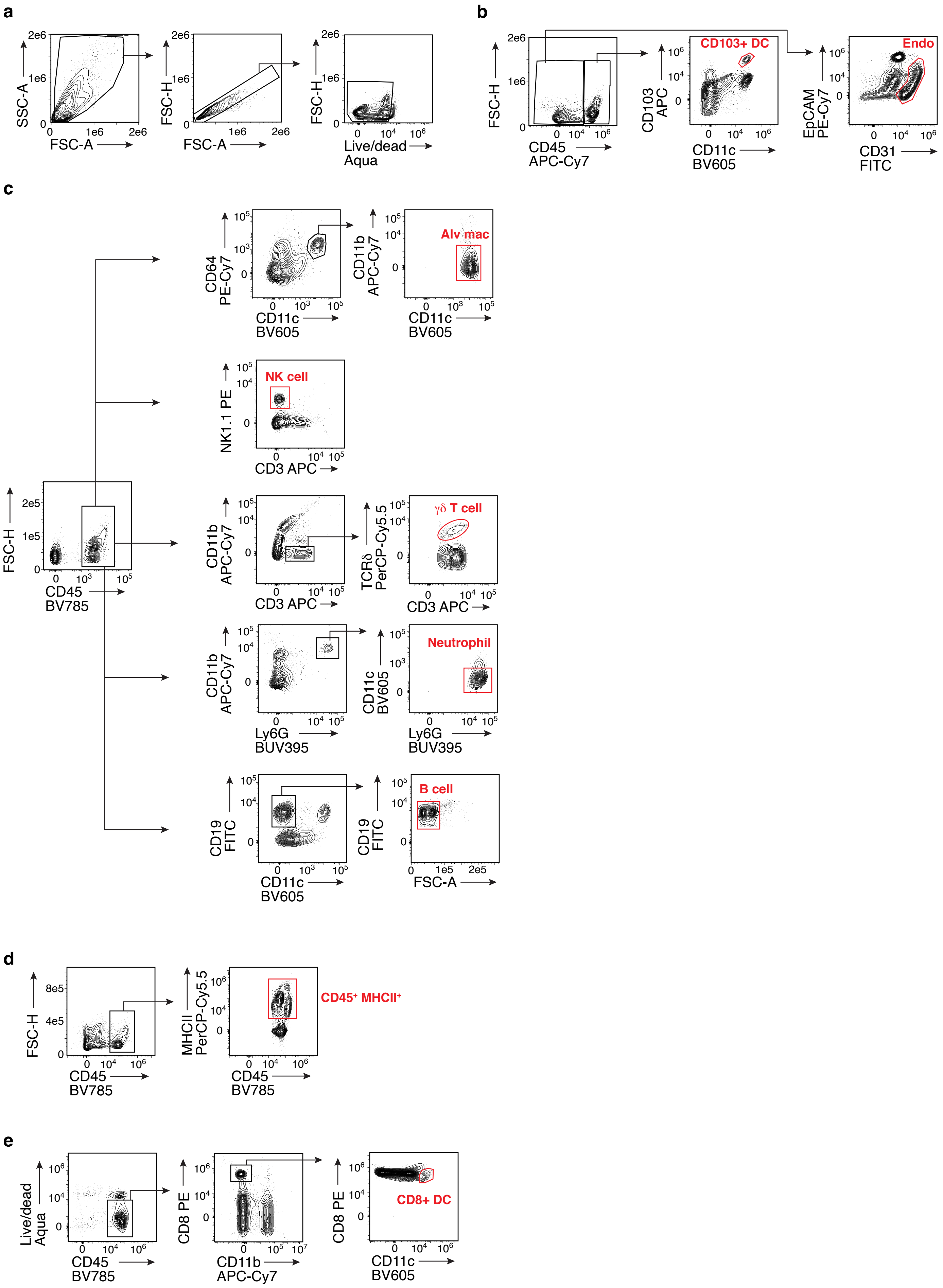

Supplementary Figure 10: Overall cell type flow cytometry gating strategies.

a, Overall example gating strategy to exclude debris, doublets, and dead cells. These gates precede those shown in all subsequent panels (**b-e**) of this figure, as well as the bulk splenocyte and BMDC populations in Supplementary Figure 5e,f. **b**, Example gating strategies for CD103$^+$ DCs (CD45$^+$, CD11c$^+$, CD103$^+$) and endothelial cells (Endo) (CD45$^-$, CD31$^+$) used in flow cytometry analyses (Figures 1b,c,f,g; 2b; 3c,d; Supplementary Figure 5d). **c,** Example gating strategies for alveolar macs (Alv mac) (CD45$^+$, CD11c$^+$, CD11b$^-$, CD64$^+$), NK cells (CD45$^+$, CD3$^-$, NK1.1$^+$), $\gamma\delta$ T cells (CD45$^+$, CD3$^+$, CD11b$^-$, TCRδ^+), neutrophils (CD45$^+$, CD11c$^-$, CD11b$^+$, Ly6G$^+$), and B cells (FSC-A^{low}, CD45$^+$, CD11c$^-$, CD19$^+$) used in flow cytometry analyses (Figures 1b; 2b; 2d,e; 3c,d; 6b-e,g; Supplementary Figure 5d). **d**, Example gating strategy for CD45$^+$ MHCII$^+$ cells (CD45$^+$, MHCII$^+$) analyses (Figure 2e). **e**, Example gating strategy for CD8$^+$ DCs (CD45$^+$, CD11b$^-$, CD11c^{hi}, CD8$^+$) analyses (Figures 3c,d). **a-e**, These gating strategies are representative examples of how the cell populations were gated using cell surface markers, dead cell exclusion, and light scatter properties. Between experiments, exact cytometer voltages/gains were subject to variation, and the precise fluorophores used for each marker sometimes differed from those displayed here depending on the other markers and fluorophores used within the same experiment. For a full list of antibody-fluorophore conjugates, see Supplementary Table 6.

Supplementary Figure 11

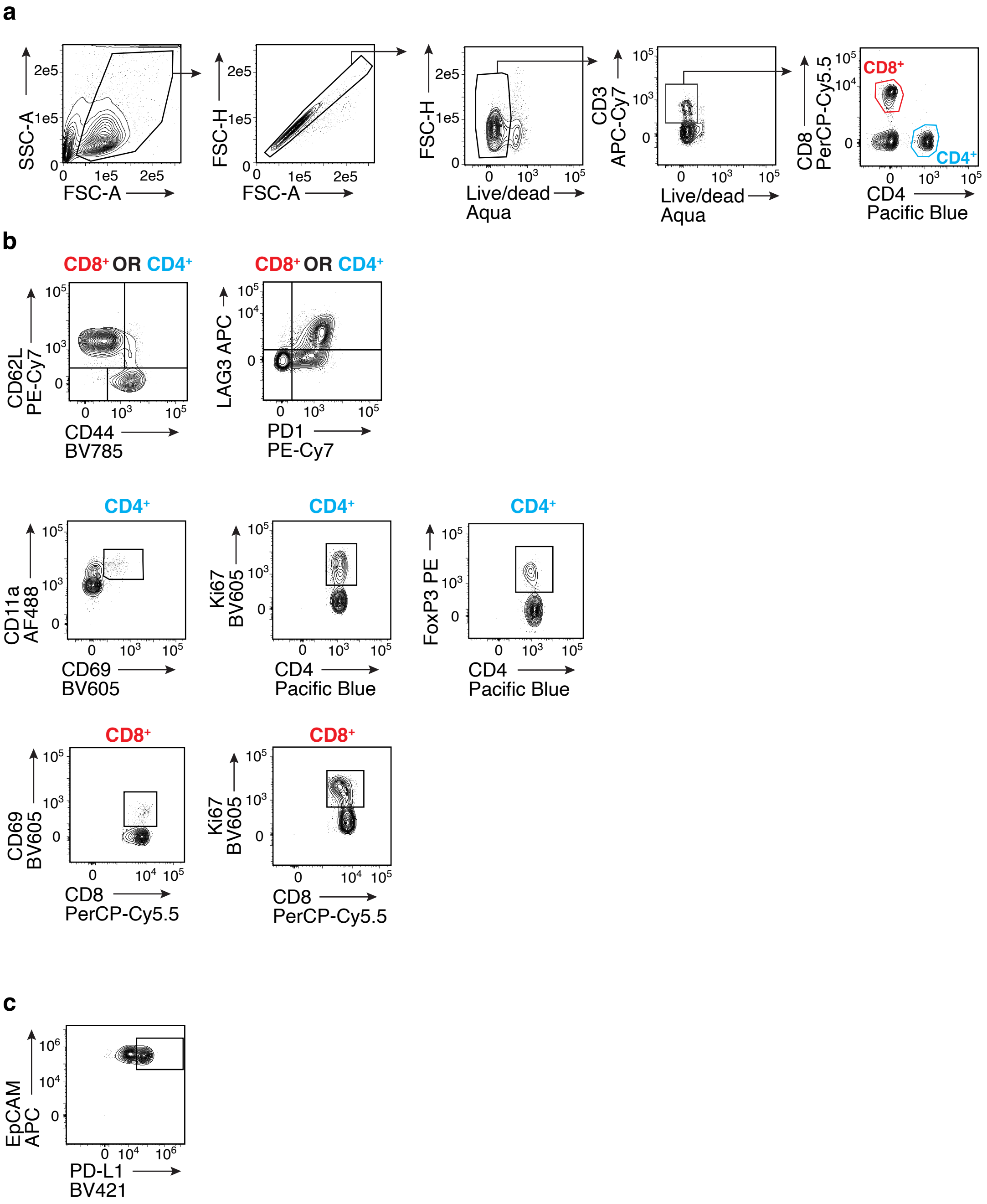

Supplementary Figure 11: Flow cytometry gating strategies for T cell subsets, activation, inhibitory, and memory markers, and AT2 PD-L1 expression.
a, Overall example gating strategy to exclude debris, doublets, dead cells, and to gate on $CD4^+$ T cells (blue; $CD3^+$, $CD4^+$, $CD8^-$) and $CD8^+$ T cells (red; $CD3^+$, $CD8^+$, $CD4^-$). These gates precede those shown in panel (**b**) of this figure. **b**, Example gating strategies for: CD44, CD62L, PD1, and LAG3 expressing T cell populations (gated similarly for both CD4 and CD8 T cells); $CD69^+$ $CD11a^+$, Ki-67^+, and $FoxP3^+$ $CD4^+$ T cell populations; $CD69^+$ and Ki-67^+ $CD8^+$ T cell populations. Used in flow cytometry analyses, as in Figures 3f-i, Supplementary Figures 6, 7, 9. **c**, Example gating strategy for PD-L1 expression on AT2s flow analysis (Supplementary Figure 9e); AT2 population was gated as detailed in Supplementary Figure 1. **a-c**, These gating strategies are representative examples of how the cell populations were gated using cell surface markers, dead cell exclusion, and light scatter properties. Between experiments, exact cytometer voltages/gains were subject to variation, and the precise fluorophores used for each marker sometimes differed from those displayed here depending on the other markers and fluorophores used within the same experiment. For a full list of antibody-fluorophore conjugates, see Supplementary Table 6.

Supplementary Figure 12

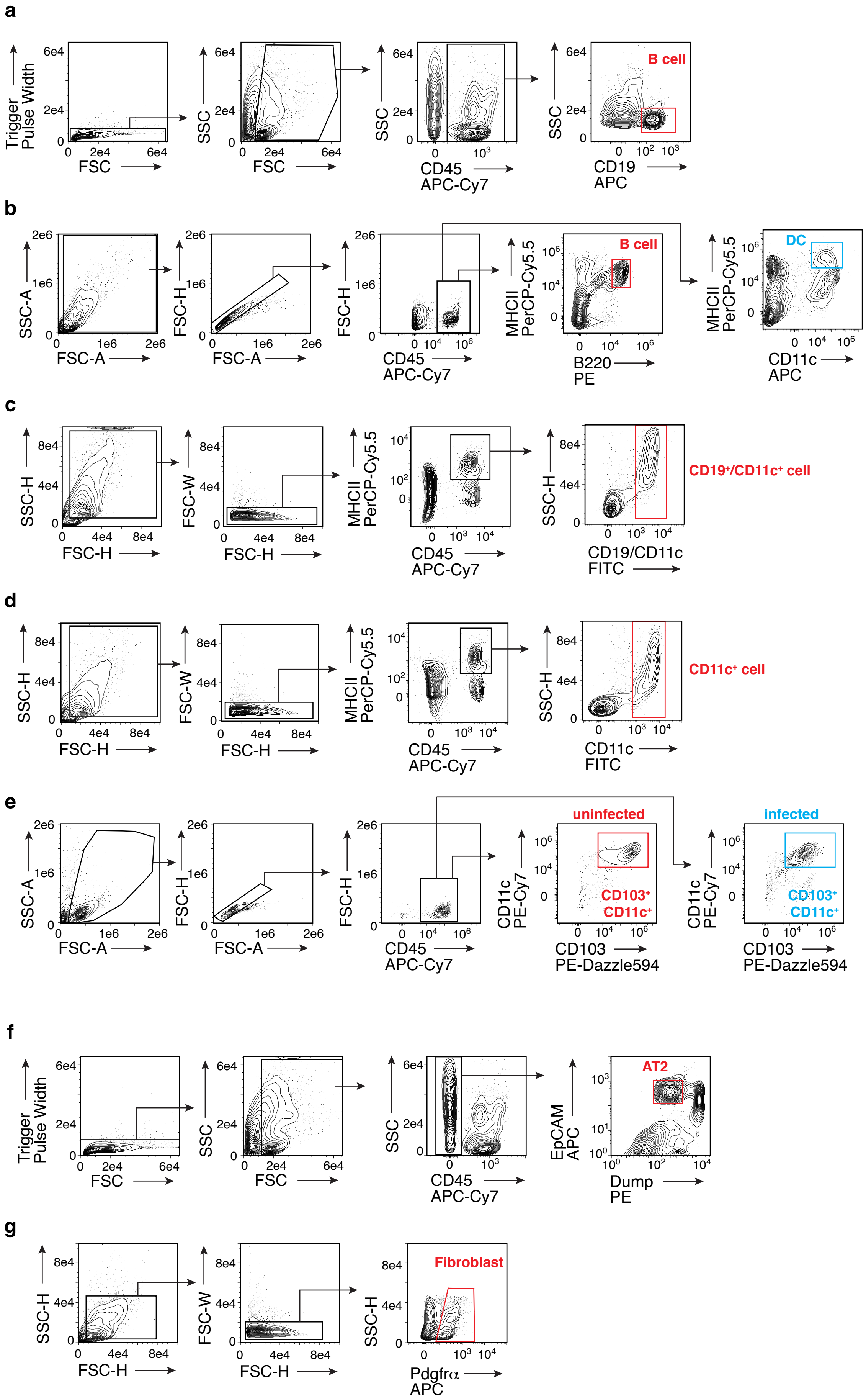

Supplementary Figure 12: Sort gating strategies for antigen presenting cell populations for presentation assays and for cells for organoid culture.

a, Example gating strategy for B cells (CD45$^+$, CD19$^+$) used in Cathepsin L assay (Supplementary Figure 5c). **b,** Example gating strategy for B cells (CD45$^+$, B220$^+$, MHCII$^+$) and DCs (CD45$^+$, CD11c$^+$, MHCII^{hi}) used in DQ-Ova assay (Figure 2a) and qPCR (Figures 1d, 2c). **c,** Example gating strategy for CD11c$^+$/ CD19$^+$ APCs (CD45$^+$, MHCII$^+$, CD11c$^+$ or CD19$^+$) used in B6 hybridoma assay (Figure 5b). **d,** Example gating strategy for CD11c$^+$ APCs (CD45$^+$, MHCII$^+$, CD11c$^+$) used in Balb/c hybridoma assay (Figure 5c). **e,** Example gating strategy for CD11c$^+$ CD103$^+$ APCs (CD45$^+$, CD11c$^+$, CD103$^+$) from uninfected (red) and infected (blue) mice used in ELIspot assay (Figure 5e); representative post-sort samples are displayed here. **f**, Example gating strategy for AT2s (CD45$^-$, CD31$^-$, Podoplanin$^-$, CD34$^-$, Sca1$^-$, EpCAM^{int})[1] sorted for organoid culture (Supplementary Figure 8). **g**, Example gating strategy for Fibroblasts (Pdgfrα^+)[1] sorted for organoid culture (Supplementary Figure 8). **a-g**, These gating strategies are representative examples of how the cell populations were gated using cell surface markers, dead cell exclusion, and light scatter properties. Between experiments, exact cytometer voltages/gains were subject to variation, and the precise fluorophores used for each marker sometimes differed from those displayed here depending on the other markers and fluorophores used within the same experiment. For a full list of antibody-fluorophore conjugates, see Supplementary Table 6.

Supplementary Figure 13

a

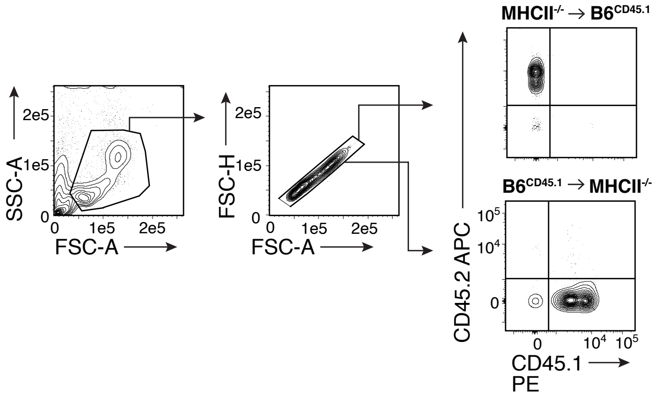

b

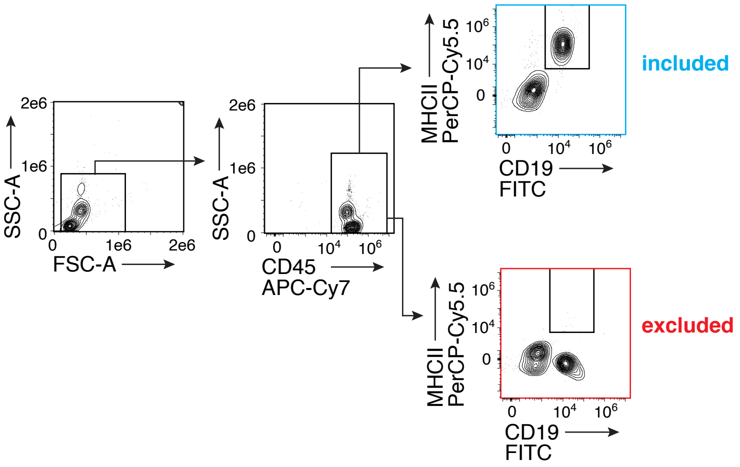

Supplementary Figure 13: Gating strategies for bone marrow chimera reconstitution analysis and AT2 MHCII conditional knockout mice germline deletion screening.
a, Example gating strategy for peripheral blood cells to exclude debris and doublets, then to assess CD45.1 or CD45.2 expression in bone marrow chimeric mice (Figure 1e). Shown are examples from 2 transfers: MHCII$^{-/-}$ → B6$^{CD45.1}$ and B6$^{CD45.1}$ → MHCII$^{-/-}$**. b,** Example gating strategy for peripheral blood cells to exclude debris and doublets, then to assess MHCII expression on B cells (CD45^{+} CD19^{+}). This peripheral blood screening was performed on all SPC$^{\Delta Ab1}$ and Ab1$^{fl/fl}$ mice used in our studies. **a-b**, These gating strategies are representative examples of how the cell populations were gated using cell surface markers and light scatter properties. Between experiments, exact cytometer voltages/gains were subject to variation, and the precise fluorophores used for each marker sometimes differed from those displayed here depending on the other markers and fluorophores used within the same experiment. For a full list of antibody-fluorophore conjugates, see Supplementary Table 6.

Supplementary Table 1

Ab1$^{fl/fl}$ vs SPC$^{\Delta Ab1}$ Comparison Results		
**Cell type**	***P*-value**	***t* or *U*, df**
Lung CD4 #	0.0435	U = 20
Lung CD8 #	0.1623	t = 1.461, 17
Lung CD4 %CD44$^+$ CD62L$^-$	0.0343	t = 2.301, 17
Lung CD4 %CD62L$^+$ CD44$^-$	0.0117	t = 2.825, 17
Lung CD8 %CD44$^+$ CD62L$^-$	0.2716	t = 1.136, 17
Lung CD8 %CD62L$^+$ CD44$^-$	0.1537	t = 1.493, 17
Lung CD4 %Foxp3$^+$	0.8810	t = 0.1519, 17
Lung CD4 %CD69$^+$ CD11a$^+$	0.2598	t = 1.166, 17
Lung CD8 %CD69$^+$	0.3186	t = 1.027, 17
Lung CD4 %PD1$^+$	0.0029	t =3.469, 17
Lung CD8 %PD1$^+$	0.0057	U = 12
Lung CD4 %Ki67$^+$	0.1728	t =1.423, 17
Lung CD8 %Ki67$^+$	0.7005	t =0.3912, 17
Alv Macs #	0.0947	U = 24
Alv Macs %	0.2348	t = 1.232, 17
Neutrophils #	0.5490	U = 37
Neutrophils %	0.6748	U = 39.5
B cells #	0.3293	t = 1.004, 17
B cells %	0.3067	t = 1.054, 17
NK cells #	0.5598	t = 0.5948, 17
NK cells %	0.3562	U = 33
γδT cells #	0.0947	U = 24
γδT cells %	0.2766	U = 31
Spleen CD4 #	0.2110	U = 29
Spleen CD8 #	0.3562	U = 33
Spleen CD4 %CD44$^+$ CD62L$^-$	0.8624	t = 0.1759, 17
Spleen CD4 %CD62L$^+$ CD44$^-$	0.5180	t = 0.6601, 17
Spleen CD8 %CD44$^+$ CD62L$^-$	0.2746	t = 1.129, 17
Spleen CD8 %CD62L$^+$ CD44$^-$	0.4660	t = 0.7458, 17
Spleen CD4 %Foxp3$^+$	0.3922	t = 0.8779, 17
Spleen CD4 %PD1$^+$	0.5021	t = 0.6857, 17
Spleen CD8 %PD1$^+$	0.1366	t = 1.563, 17
Spleen CD4 %Ki67$^+$	0.3096	t = 1.047, 17
Spleen CD8 %Ki67$^+$	0.6752	t = 0.4263, 17

Supplementary Table 1: Statistical test results for comparisons of lung and spleen immune cells at homeostasis in Ab1$^{fl/fl}$ and SPC$^{\Delta Ab1}$ mice.

Results of unpaired two-tailed Student's *t*-tests and Mann-Whitney tests comparing immune cell populations between Ab1$^{fl/fl}$ and SPC$^{\Delta Ab1}$ mice. *P* values and associated *U* or *t* statistics with associated degrees of freedom (df) for each comparison are reported. Statistical results in this table are for the data depicted in Figures 3f-i and Supplementary Figure 6.

Supplementary Table 2

Ab1$^{fl/fl}$ vs SPC$^{\Delta Ab1}$ Comparison Results			
**Experiment**	**Cell type**	***P*-value**	***t*, df**
Homeostasis analysis	Lung CD4 %LAG3^{+}	0.6918	*t* = 0.4032, 17
	Lung CD8 %LAG3^{+}	0.3662	*t* =0.9285, 17
	Spleen CD4 %LAG3^{+}	0.4868	*t* = 0.7109, 17
	Spleen CD8 %LAG3^{+}	0.6578	*t* = 0.4508, 17
9 days post IAV infection	Lung CD4 %LAG3^{+}	0.8912	*t* = 0.1403, 17
	Lung CD4 #LAG3^{+}	0.9855	*t* = 0.01860, 17
	Lung CD8 %LAG3^{+}	0.4240	*t* = 0.8336, 17
	Lung CD8 #LAG3^{+}	0.7958	*t* = 0.2658, 17

Supplementary Table 2: Statistical test results for comparisons of LAG3 expressing T cells in Ab1$^{fl/fl}$ and SPC$^{\Delta Ab1}$ mice.
Results of unpaired two-tailed Student's *t*-tests and Mann-Whitney tests comparing LAG3-expressing T cell populations between Ab1$^{fl/fl}$ and SPC$^{\Delta Ab1}$ mice. *P* values and associated *U* or *t* statistics with associated degrees of freedom (df) for each comparison are reported. Statistical results in this table are for the data depicted in Supplementary Figure 7.

Supplementary Table 3

Overall model effects		
**Factor**	***P*-value**	***F (*df.n, df.d)**
Time	<0.0001	177.4 (2.020, 188.5)
Genotype	0.1696	1.916 (1, 94)
Genotype x Time	0.0002	3.476 (10, 933)

Post-hoc comparison results by day		
**Day post-infection**	***P*-value**	***t*, df**
5	>0.9999	0.08273, 93.52
6	>0.9999	0.4987, 91.57
7	0.9995	0.6777, 90.66
8	>0.9999	0.06608, 81.36
9	>0.9999	0.2905, 82.35
10	0.9870	0.9875, 84.87
11	0.8544	1.414, 91.06
12	0.7380	1.593, 92.94
13	0.3238	2.141, 91.81
14	0.6217	1.744, 92.33
15	0.5448	1.839, 92.95

Supplementary Table 3: Mixed-effects model analysis results for weight loss after IAV infection. Results of repeated measures mixed-effects model (REML) statistical analysis with Geisser-Greenhouse correction factoring in both genotype and day post IAV infection as variables, as well as results of post-hoc multiple comparisons with Sidak's correction between the strains at each time point post infection. *P* values and associated *F* or *t* statistics with associated degrees of freedom (df) for the overall effects as well as for each post-hoc comparison are reported, calculated based on weights expressed as a percentage of initial weight. Data depicted in this table are in Figure 4a.

Supplementary Table 4

Overall model effects		
**Factor**	***P*-value**	***F* (df.n, df.d)**
Time	<0.0001	145.9 (2.652, 87.69)
Genotype	0.0037	9.582 (1, 37)
Genotype x Time	<0.0001	3.707 (14, 463)

Post-hoc comparison results by day		
**Day post-infection**	***P*-value**	***t*, df**
2	0.5271	2.051, 31.28
4	0.0950	2.878, 36.78
5	0.1109	2.818, 35.97
6	0.5318	2.033, 36.94
7	0.4242	2.175, 36.79
8	0.7410	1.767, 34.18
9	0.4465	2.153, 33.4
10	0.0429	3.215, 32.91
11	0.2627	2.447, 31.76
12	0.6094	1.95, 29.34
13	0.4114	2.221, 27.74
14	0.3520	2.325, 24.9
15	0.2886	2.441, 23.79
16	0.4587	2.16, 26.21
17	0.5085	2.1, 24.41

Supplementary Table 4: Mixed-effects model analysis results for weight loss after SeV infection. Results of repeated measures mixed-effects model (REML) statistical analysis with Geisser-Greenhouse correction factoring in both genotype and day post SeV infection as variables, as well as results of post-hoc multiple comparisons with Sidak's correction between the strains at each time point post infection. *P* values and associated *F* or *t* statistics with associated degrees of freedom (df) for the overall effects as well as for each post-hoc comparison are reported, calculated based on weights expressed as a percentage of initial weight. Data depicted in this table are in Figure 4b.

Supplementary Table 5

Ab1$^{fl/fl}$ vs SPC$^{\Delta Ab1}$ Comparison Results			
**Experiment**	**Cell type**	***P*-value**	***t* or *U*, df**
Lung T cell analysis	# CD4	0.2933	t = 1.073, 26
	CD4 %CD44^{+} CD62L^{-}	0.5433	t = 0.6176, 26
	CD4 %CD62L^{+} CD44^{-}	0.8360	t = 0.2091, 26
	# CD4 PD1^{+}	0.3491	t = 0.9535, 26
	CD4 %PD1^{+}	0.4146	t = 0.8291, 26
	# CD8 PD1^{+}	0.8229	t = 0.2261, 26
	CD8 %PD1^{+}	0.7667	t = 0.2998, 26
PD-L1 expression analysis	AT2s %PD-L1^{+}	0.6806	U = 27

Supplementary Table 5: Statistical test results for comparisons of PD-L1 expressing AT2s and PD1, CD44, and CD62L expressing T cells in IAV infected Ab1$^{fl/fl}$ and SPC$^{\Delta Ab1}$ mice.
Results of unpaired two-tailed Student's *t*-tests, Welch's *t*-test, and Mann-Whitney test, comparing PD-L1-expressing AT2 cells, as well as bulk CD4^{+} T cells, CD44/CD62L CD4^{+} T cells, and PD1-expressing T cell populations between Ab1$^{fl/fl}$ and SPC$^{\Delta Ab1}$ mice 9 days post IAV infection. *P* values and associated *U* or *t* statistics with associated degrees of freedom (df) for each comparison are reported. Statistical results in this table are for the data depicted in Supplementary Figure 9.

Supplementary Table 6

Flow cytometry antibodies used					
**Target**	**Clone**	**Source**	**Catalog #**	**Fluorophores**	**Dilution**
mouse CD45	30-F11	Biolegend	103116; 103149	APC-Cy7; BV785	1:100
mouse CD31	390	Biolegend	102408; 102406	PE; FITC	1:100
mouse EpCAM (CD326)	G8.8	Biolegend	118216; 118214	PE-Cy7; APC	1:100
mouse I-A/I-E (MHCII)	M5/114.15.2	Biolegend; Thermo Fisher	107626; 48-5321-82	PerCP-Cy5.5; eFluor450	1:100
mouse proSP-C	AB3786	Sigma Aldrich	AB3786	unconjugated	1:100
rabbit IgG	Poly4064	Biolegend	406414	AF647	1:100
human HT2-280	TB-27AHT2-280	Terrace Biotech	TB-27AHT2-280	unconjugated	1:50
mouse IgM	II/41	Thermo Fisher	46-5790-80	PerCP-eFluor710	1:100
mouse CD11c	N418	Biolegend	117334; 117318; 117306; 117310	BV605; PE-Cy7; FITC; APC	1:100
mouse CD103	2E7	Biolegend	121414; 121430	APC; PE-Dazzle594	1:100
mouse CD11b	M1/70	BD; Biolegend	557657; 101208	APC-Cy7; PE	1:100
mouse CD64	X54-5/7.1	Biolegend	139314	PE-Cy7	1:100
mouse CD3	145-2C11	BD; Biolegend	557596; 100362	APC-Cy7; APC-Fire750	1:100
mouse CD3	17A2	Biolegend	100203; 100236	FITC; APC	1:100
mouse CD8	53-6.7	BD; Biolegend	551162; 100707	PerCP-Cy5.5; PE	1:100
mouse CD19	6D5	Biolegend	115505; 115511	FITC; APC	1:100
mouse B220	RA3-6B2	Biolegend	103207	PE	1:100
mouse CD4	RM4-5	Biolegend; BD	100531; 563727	PacBlue; BV785	1:100
mouse NK1.1	PK136	Thermo Fisher	12-5941-81	PE	1:100
mouse TCRd	GL3	Biolegend	118118	PerCP-Cy5.5	1:100
mouse Ly6G	1A8	BD	563978	BUV395	1:100
mouse Podoplanin	8.1.1	Thermo Fisher	12-5381-82	PE	1:100
mouse Sca-1	D7	Thermo Fisher	12-5981-82	PE	1:100
mouse CD34	MEC14.7	Biolegend	119308	PE	1:100
mouse Pdgfra (CD140a)	APA5	Biolegend	135907	APC	1:250

Supplementary Table 6 (cont'd)

Flow cytometry antibodies used, continued					
**Target**	**Clone**	**Source**	**Catalog #**	**Fluorophores**	**Dilution**
human HLA-DR	G46-6	BD	562844	BV605	1:100
mouse I-A^b	AF-120.1	Thermo Fisher	48-5320-80	eFluor450	1:100
mouse I-E^d	14-4-4S	Biolegend	110211	AF647	1:100
mouse invariant chain (CD74)	In-1	BD	740274	BUV395	1:100
mouse H2-Mab2	2E5A	BD	552405	unconjugated	1:100
mouse H2-Ob	Mags.Ob1	L. Denzin	N/A	AF647	1:500
human invariant chain (CD74)	Pin.1	Biolegend	357609	PE-Cy7	1:500
human HLA-DM	Map.DM1	L. Denzin	N/A	AF488	1:500
mouse CLIP/I-A^b	15G4	L. Denzin	N/A	AF647	1:500
mouse Ea^{52-68}/I-A^b	YAe	Thermo Fisher	13-5741-81	Biotin	1:50
mouse CD80	16-10A1	BD	560526	PerCP-Cy5.5	1:50
mouse CD86	GL1	BD	560581	AF700	1:50
mouse ICAM-1 (CD54)	YN1/1.7.4	Biolegend	116123	PerCP-Cy5.5	1:50
mouse CD45.1	A20	Biolegend	110708	PE	1:100
mouse CD45.2	104	Biolegend	109814	APC	1:100
influenza hemagglutinin (HA)	IC5-4F8	BEI	NR-48783	unconjugated	1:50
influenza nucleoprotein (NP)	D67J	Thermo Fisher	MA1-7322	FITC	1:100
mouse CD44	IM7	Biolegend	103059; 103006	BV785; FITC	1:100
mouse CD62L	MEL-14	Biolegend	104417; 104426	PE-Cy7; AF700	1:100
mouse CD69	H1.2F3	Biolegend	104530	BV605	1:100
mouse CD11a	M17/4	Biolegend	101111	AF488	1:100
mouse PD1	RMP1-30	Biolegend	109110	PE-Cy7	1:100
mouse LAG3	C9B7W	Biolegend	125210	APC	1:100
mouse Foxp3	FJK-16s	Thermo Fisher	12-5773-82	PE	1:100
mouse Ki67	16A8	Biolegend	652413	BV605	1:100
mouse PD-L1	MIH5	BD	564716	BV421	1:100

Supplementary Table 6: List of flow cytometry antibodies used.

Supplementary Table 7

RT-PCR primers used	
**Target**	**Sequence**
H2-Aa-F	CTGATTCTGGGGGTCCTCGC
H2-Aa-R	CCTACGTGGTCGGCCTCAAT
H2-Ab1-F	GAGCAAGATGTTGAGCGGCA
H2-Ab1-R	GCCTCGAGGTCCTTTCTGACTC
H2-DMa-F	GGCGGTGCTCGAAGCA
H2-DMa-R	TGTGCCGGAATGTGTGGTT
H2-DMb1-F	CTATCCAGCGGATGTGACCAT
H2-DMb1-R	TGGGCTGAGCCGTCTTCT
Hprt1-F	TCAGTCAACGGGGGACATAA
Hprt1-R	GGGGCTGTACTGCTTAACCAG

Supplementary Table 7: List of primers used for qPCR.

Supplementary References

1. Paris, A.J. *et al.* STAT3-BDNF-TrkB signalling promotes alveolar epithelial regeneration after lung injury. *Nat Cell Biol* **22**, 1197-1210 (2020).

2. Ma, J.Z. *et al.* Unique Transcriptional Architecture in Airway Epithelial Cells and Macrophages Shapes Distinct Responses following Influenza Virus Infection Ex Vivo. *J Virol* **93** (2019).
