# Supplementary material for: Type II alveolar cell MHCII improves respiratory viral disease outcomes while exhibiting limited antigen presentation
Source: Nat Commun. 2021 Jun 28;12:3993. doi: 10.1038/s41467-021-23619-6 (PMC8239023; doi:10.1038/s41467-021-23619-6)
Supplement: Supplementary file 3 — Reporting Summary [file 41467_2021_23619_MOESM3_ESM.pdf]

nature research

Corresponding author(s): Laurence C. Eisenlohr

Last updated by author(s): Apr 17, 2021

Reporting Summary

Nature Research wishes to improve the reproducibility of the work that we publish. This form provides structure for consistency and transparency in reporting. For further information on Nature Research policies, see our Editorial Policies and the Editorial Policy Checklist.

Statistics

For all statistical analyses, confirm that the following items are present in the figure legend, table legend, main text, or Methods section.

n/a	Confirmed	
☐	☒	The exact sample size (*n*) for each experimental group/condition, given as a discrete number and unit of measurement
☐	☒	A statement on whether measurements were taken from distinct samples or whether the same sample was measured repeatedly
☐	☒	The statistical test(s) used AND whether they are one- or two-sided<br>*Only common tests should be described solely by name; describe more complex techniques in the Methods section.*
☐	☒	A description of all covariates tested
☐	☒	A description of any assumptions or corrections, such as tests of normality and adjustment for multiple comparisons
☐	☒	A full description of the statistical parameters including central tendency (e.g. means) or other basic estimates (e.g. regression coefficient) AND variation (e.g. standard deviation) or associated estimates of uncertainty (e.g. confidence intervals)
☐	☒	For null hypothesis testing, the test statistic (e.g. *F*, *t*, *r*) with confidence intervals, effect sizes, degrees of freedom and *P* value noted<br>*Give P values as exact values whenever suitable.*
☒	☐	For Bayesian analysis, information on the choice of priors and Markov chain Monte Carlo settings
☒	☐	For hierarchical and complex designs, identification of the appropriate level for tests and full reporting of outcomes
☒	☐	Estimates of effect sizes (e.g. Cohen's *d*, Pearson's *r*), indicating how they were calculated

Our web collection on statistics for biologists contains articles on many of the points above.

Software and code

Policy information about availability of computer code

Data collection	Flow cytometry data from LSRII and LSRFortessa cytometers was acquired with FACSDiva software (BD), version 8.0.2. Flow cytometry data from Cytoflex S and LX cytometers was acquired with CytExpert software (Beckman Coulter), version 2.4. Data from cell sorting performed on the FACSAria Fusion sorter was acquired with FACSDiva software (BD), version 8.0.2. Data from cell sorting performed on the FACSJazz sorter was acquired with FACS Sortware software (BD), version 1.1.0.84. Data from cell sorting performed on the MoFlo Astrios sorter was acquired with Summit software (Beckman Coulter), version 6.3.1.<br><br>Immunofluorescence images were captured on the Axio Observer 7 widefield microscope with Axiocam 702 monochrome CMOS camera using ZEN 2.5 (blue edition) acquisition software (Zeiss). Organoid images were captured on the EVOS FL Auto using built-in EVOS FL Auto software (Thermo Fisher), Software Revision 31201. H&E images were acquired using an Aperio AT2 Slide Scanner (Leica).<br><br>qPCR data were acquired on a StepOnePlus Real Time PCR Machine using StepOnePlus software (Applied Biosystems), version 2.1. Fluorescence measurements were taken using an Infinite M200 Pro Plate Reader with i-control 2.0 software (Tecan). ELISpot plate images were captured and spots counted using the CTL ImmunoSpot S6 Universal Analyzer with ImmunoSpot software (ImmunoSpot); analyzer version 5.2, counting version 6.0, and capture version 6.6.
Data analysis	Flow cytometry data were analyzed using FlowJo (V10.6.2). H&E images were evaluated using Aperio ImageScope software (Leica), version 12.4.5. Immunofluorescence and organoid images were processed and analyzed in FIJI ImageJ (V1.0). Statistical analyses were performed using GraphPad Prism (V8.4.2). General data documentation was performed in Microsoft Excel (V16.37). Final images were composed for publication in Adobe Illustrator (V24.1). Model diagrams were created in Adobe Illustrator (V24.1), modeled partially after cartoons originally from BioRender, accessed with a paid subscription that provides permission for publication. RNA-sequencing data were analyzed using Salmon v1.2.1 and R v4.0.2 with the biomaRt v3.12 package, as described in the Methods.

For manuscripts utilizing custom algorithms or software that are central to the research but not yet described in published literature, software must be made available to editors and reviewers. We strongly encourage code deposition in a community repository (e.g. GitHub). See the Nature Research guidelines for submitting code & software for further information.

Data

Policy information about availability of data

All manuscripts must include a data availability statement. This statement should provide the following information, where applicable:

- Accession codes, unique identifiers, or web links for publicly available datasets
- A list of figures that have associated raw data
- A description of any restrictions on data availability

All original data generated in this study are either available within the paper and its supplementary information files or are available from the corresponding author upon reasonable request. Source data are also provided with the paper as a separate Source Data file. The FASTQ files used in our RNA-sequencing analysis were originally generated by Ma et. al., J Virol, 2019; they are publicly available via the GEO accession viewer as GSE 115904 and can be found at the following URL: https://www.ncbi.nlm.nih.gov/geo/query/acc.cgi?acc=GSE115904.

Field-specific reporting

Please select the one below that is the best fit for your research. If you are not sure, read the appropriate sections before making your selection.

☒ Life sciences ☐ Behavioural & social sciences ☐ Ecological, evolutionary & environmental sciences

For a reference copy of the document with all sections, see nature.com/documents/nr-reporting-summary-flat.pdf

Life sciences study design

All studies must disclose on these points even when the disclosure is negative.

Sample size	Sample sizes were chosen based on past experience of the laboratory (Miller et al., Nat. Med., 2015) and standard practices. For in vivo infection experiments, minimum sample sizes were based on prior publications using in vivo influenza and Sendai virus models (Miller et al., Nat. Med., 2015; Linderman et al., PLoS Path, 2016; Tapia et al., PLoS Path, 2013); larger sample sizes were often used to account for the variability expected with in vivo infections, and as mice of the Ab1fl/fl background exhibit germline recombination at ~5% based on prior publications (Mundt et al., Sci Immunol, 2019). Where large biological sample sizes were prohibitive due to technical limitations (ie: cell sorting-dependent experiments), small biological samples were used in multiple independent experiments. No statistical calculations were used to determine sample size a priori. Sample sizes are indicated in each figure legend.
Data exclusions	No data were excluded.
Replication	As reported in detail in the figure legends, experiments were performed multiple times and/or with multiple biological replicates. All attempts at replication were successful.
Randomization	For all studies examining the impact of the loss of AT2 MHCII, mice were not randomized into groups as mouse genotype was a critical variable measured in our studies; thus, mice were intentionally age and sex matched across genotypes for all such experiments and cohoused whenever possible. For studies in which treatment effect was a variable (such as IFNg administration), WT mice (all of similar age/sex) were randomly assigned to each group (PBS v IFNg treatment). For in vitro studies, randomization was irrelevant to the experimental design and was therefore not performed.
Blinding	For homeostasis and infection studies comparing conditional knockout mice to controls, after genotypes were recorded, mice were referred to by cage number and ear tag and/or an overall study ID number, instead of by genotype, in order to maintain blinding during data acquisition and analysis. H&E slide lung pathology interpretation was performed in a blinded manner by a veterinary pathologist. In all other mouse studies, blinding was not performed as genotype and/or in vivo treatment were critical variables that required tracking as well as careful cage labeling and genotype/ treatment-specific mouse monitoring. For in vitro experiments, blinding was not performed as careful plate/tube labeling was required to keep track of multiple simultaneous experimental conditions.

Reporting for specific materials, systems and methods

We require information from authors about some types of materials, experimental systems and methods used in many studies. Here, indicate whether each material, system or method listed is relevant to your study. If you are not sure if a list item applies to your research, read the appropriate section before selecting a response.

Materials & experimental systems

n/a	Involved in the study
☐	☒ Antibodies
☐	☒ Eukaryotic cell lines
☒	☐ Palaeontology and archaeology
☐	☒ Animals and other organisms
☐	☒ Human research participants
☒	☐ Clinical data
☒	☐ Dual use research of concern

Methods

n/a	Involved in the study
☒	☐ ChIP-seq
☐	☒ Flow cytometry
☒	☐ MRI-based neuroimaging

Antibodies

Antibodies used

Information for all flow cytometry antibodies used in the study, including target, clone name, catalog number, supplier, and dilution used, are listed in Supplementary Table 6. All antibodies used for immunofluorescence are listed in the methods section.

Validation

In all flow cytometry and IF experiments, positive control samples were used to confirm adequate antibody target binding within every experiment. Negative controls were also used in every experiment to account for non-specific staining; true biologically negative samples were used whenever possible, but if unavailable, fluorescence-minus-one or unstained controls were used instead. In Ag-primary + secondary-fluorophore staining combinations, secondary-fluorophore-only conditions were used as additional controls for further validation and to account for any nonspecific staining.

For the anti-mouse CLIP/I-Ab antibody 15G4 obtained from L. Denzin, validation was first published in Liljedahl et al., Immunity, 1998.
For the anti-human HLA-DM antibody Map.DM1 obtained from L. Denzin, validation was first published in Hammond et al., J Immunol, 1998.
For the anti-mouse H2-Ob antibody Mags.Ob1 obtained from L. Denzin, validation was first published in Fallas et al., J Immunol, 2007.

All commercially available antibodies have been validated by the supplier for the indicated target and application, with relevant publications and validation information available on the manufacturer's websites for each antibody. Suppliers used are: Biolegend, BD, BEI, Sigma Aldrich, Thermo Fisher, Terrace Biotech, EMD Millipore, and Abcam, and validation information can be found on the website corresponding to each specific antibody by using the catalog number listed in Supplementary Table 6 or the methods section. The URLs are also listed below.

CD45 APC-Cy7: https://www.biolegend.com/en-us/products/apc-cyanine7-anti-mouse-cd45-antibody-2530
CD45 BV785: https://www.biolegend.com/en-us/products/brilliant-violet-785-anti-mouse-cd45-antibody-10636
CD31 PE: https://www.biolegend.com/en-us/products/pe-anti-mouse-cd31-antibody-122
CD31 FITC: https://www.biolegend.com/en-us/search-results/fitc-anti-mouse-cd31-antibody-120
EpCAM PE-Cy7: https://www.biolegend.com/en-us/products/pe-cyanine7-anti-mouse-cd326-ep-cam-antibody-5303
EpCAM APC: https://www.biolegend.com/en-us/search-results/apc-anti-mouse-cd326-ep-cam-antibody-4974
MHCII PerCP-Cy5.5: https://www.biolegend.com/en-us/products/percp-cyanine5-5-anti-mouse-i-a-i-e-antibody-4282
MHCII eFluor450: https://www.thermofisher.com/antibody/product/MHC-Class-II-I-A-I-E-Antibody-clone-M5-114-15-2-Monoclonal/48-5321-82
pro-SPC unconj: https://www.sigmaaldrich.com/catalog/product/mm/ab3786
Rb IgG AF647: https://www.biolegend.com/en-us/products/alexa-fluor-647-donkey-anti-rabbit-igg-minimal-x-reactivity-9379
HT2-280 unconj: https://www.terracebiotech.com/product-page/anti-ht2-280-1ml
Mo IgM PerCP-eFluor710: https://www.thermofisher.com/antibody/product/IgM-Antibody-clone-II-41-Monoclonal/46-5790-80
CD11c BV605: https://www.biolegend.com/en-us/products/brilliant-violet-605-anti-mouse-cd11c-antibody-7865
CD11c PE-Cy7: https://www.biolegend.com/en-us/search-results/pe-cyanine7-anti-mouse-cd11c-antibody-3086
CD11c FITC: https://www.biolegend.com/en-us/search-results/fitc-anti-mouse-cd11c-antibody-1815
CD11c APC: https://www.biolegend.com/en-us/search-results/apc-anti-mouse-cd11c-antibody-1813
CD103 APC: https://www.biolegend.com/en-us/products/apc-anti-mouse-cd103-antibody-4914
CD103 PE-Dazzle594: https://www.biolegend.com/en-us/search-results/pe-dazzle-594-anti-mouse-cd103-antibody-12519
CD11b APC-Cy7: https://www.bdbiosciences.com/us/applications/research/stem-cell-research/mesenchymal-stem-cell-markers-bone-marrow/human/negative-markers/apc-cy7-rat-anti-cd11b-m170/p/557657
CD11b PE: https://www.biolegend.com/en-us/products/pe-anti-mouse-human-cd11b-antibody-349
CD64 PE-Cy7: https://www.biolegend.com/en-us/products/pe-cyanine7-anti-mouse-cd64-fcgammari-antibody-10062
CD3 APC-Cy7: https://www.bdbiosciences.com/us/applications/research/t-cell-immunology/th-1-cells/surface-markers/mouse/apc-cy7-hamster-anti-mouse-cd3e-145-2c11/p/557596
CD3 APC-Fire750: https://www.biolegend.com/en-us/products/apc-fire-750-anti-mouse-cd3epsilon-antibody-13559
CD3 FITC: https://www.biolegend.com/en-us/products/fitc-anti-mouse-cd3-antibody-45
CD3 APC: https://www.biolegend.com/en-us/products/apc-anti-mouse-cd3-antibody-8055
CD8 PerCP-Cy5.5: https://www.bdbiosciences.com/us/reagents/research/antibodies-buffers/immunology-reagents/anti-mouse-antibodies/cell-surface-antigens/percp-cy55-rat-anti-mouse-cd8a-53-67/p/551162
CD8 PE: https://www.biolegend.com/en-us/products/pe-anti-mouse-cd8a-antibody-155
CD19 FITC: https://www.biolegend.com/en-us/products/fitc-anti-mouse-cd19-antibody-1528
CD19 APC: https://www.biolegend.com/en-us/products/apc-anti-mouse-cd19-antibody-1526
B220 PE: https://www.biolegend.com/en-us/products/pe-anti-mouse-human-cd45r-b220-antibody-447

CD4 Pacific Blue: https://www.biolegend.com/en-us/products/pacific-blue-anti-mouse-cd4-antibody-2855
CD4 BV785: https://www.bdbiosciences.com/eu/applications/research/t-cell-immunology/th-1-cells/surface-markers/mouse/bv786-rat-anti-mouse-cd4-rm4-5-also-known-as-rm45/p/563727
NK1.1 PE: https://www.thermofisher.com/antibody/product/NK1-1-Antibody-clone-PK136-Monoclonal/12-5941-81
TCRd PerCP-Cy5.5: https://www.biolegend.com/en-us/products/percp-cyanine5-5-anti-mouse-tcr-gamma-delta-antibody-6702
Ly6G BUV395: https://www.bdbiosciences.com/us/reagents/research/antibodies-buffers/immunology-reagents/anti-mouse-antibodies/cell-surface-antigens/buv395-rat-anti-mouse-ly-6g-1a8/p/563978
Podoplanin PE: https://www.thermofisher.com/antibody/product/Podoplanin-Antibody-clone-eBio8-1-1-8-1-1-Monoclonal/12-5381-82
Sca-1 PE: https://www.thermofisher.com/antibody/product/Ly-6A-E-Sca-1-Antibody-clone-D7-Monoclonal/12-5981-82
CD34 PE: https://www.biolegend.com/en-us/products/pe-anti-mouse-cd34-antibody-3072
Pdgfra APC: https://www.biolegend.com/en-us/products/apc-anti-mouse-cd140a-antibody-6439
HLA-DR BV605: https://www.bdbiosciences.com/us/applications/research/stem-cell-research/mesenchymal-stem-cell-markers-bone-marrow/human/negative-markers/bv605-mouse-anti-human-hla-dr-g46-6/p/562844
I-Ab eFluor450: https://www.thermofisher.com/antibody/product/MHC-Class-II-I-Ab-Antibody-clone-AF6-120-1-Monoclonal/48-5320-80
I-Ed AF647: https://www.biolegend.com/en-us/products/alexa-fluor-647-anti-mouse-i-ek-rat-rt1d-antibody-3214
mo CD74 BUV395: https://www.bdbiosciences.com/us/reagents/research/antibodies-buffers/immunology-reagents/anti-mouse-antibodies/cell-surface-antigens/buv395-rat-anti-mouse-cd74-in-1/p/740274
H2Mab2 unconj: https://www.bdbiosciences.com/us/applications/research/intracellular-flow/intracellular-antibodies-and-isotype-controls/anti-mouse-antibodies/purified-rat-anti-mouse-h2-m-2e5a/p/552405
hu CD74 PE-Cy7: https://www.biolegend.com/en-us/products/pe-cyanine7-anti-human-cd74-cytoplasmic-antibody-15164
YAe Biotin: https://www.thermofisher.com/antibody/product/Ea52-68-peptide-bound-to-I-Ab-Antibody-clone-eBioY-Ae-YAe-Y-Ae-Monoclonal/13-5741-81
CD80 PerCP-Cy5.5: https://www.bdbiosciences.com/us/applications/research/b-cell-research/surface-markers/mouse/percp-cy55-hamster-anti-mouse-cd80-16-10a1/p/560526
CD86 AF700: https://www.bdbiosciences.com/us/applications/research/b-cell-research/surface-markers/mouse/alexa-fluor-700-rat-anti-mouse-cd86-gl1/p/560581
ICAM-1 PerCP-Cy5.5: https://www.biolegend.com/en-us/products/percp-cyanine5-5-anti-mouse-cd54-antibody-14748
CD45.1 PE: https://www.biolegend.com/en-us/products/pe-anti-mouse-cd45-1-antibody-199
CD45.2 APC: https://www.biolegend.com/en-us/products/apc-anti-mouse-cd45-2-antibody-2759
flu HA unconj: https://www.beiresources.org/Catalog/BEIMonoclonalAntibodies/NR-48783.aspx
flu NP FITC: https://www.thermofisher.com/antibody/product/Influenza-A-NP-Antibody-clone-D67J-Monoclonal/MA1-7322
CD44 BV785: https://www.biolegend.com/en-us/products/brilliant-violet-785-anti-mouse-human-cd44-antibody-7959
CD44 FITC: https://www.biolegend.com/en-us/search-results/fitc-anti-mouse-human-cd44-antibody-314
CD62L PE-Cy7: https://www.biolegend.com/en-us/products/pe-cyanine7-anti-mouse-cd62l-antibody-1922
CD62L AF700: https://www.biolegend.com/en-us/search-results/alexa-fluor-700-anti-mouse-cd62l-antibody-3409
CD69 BV605: https://www.biolegend.com/en-us/products/brilliant-violet-605-anti-mouse-cd69-antibody-7864
CD11a AF488: https://www.biolegend.com/en-us/products/alexa-fluor-488-anti-mouse-cd11a-antibody-3200
PD1 PE-Cy7: https://www.biolegend.com/en-us/products/pe-cyanine7-anti-mouse-cd279-pd-1-antibody-3612
LAG3 APC: https://www.biolegend.com/en-us/products/apc-anti-mouse-cd223-lag-3-antibody-6926
FoxP3 PE: https://www.thermofisher.com/antibody/product/FOXP3-Antibody-clone-FJK-16s-Monoclonal/12-5773-82
Ki67 BV605: https://www.biolegend.com/en-us/products/brilliant-violet-605-anti-mouse-ki-67-antibody-8983
PDL1 BV421: https://www.bdbiosciences.com/us/applications/research/b-cell-research/surface-markers/mouse/bv421-rat-anti-mouse-cd274-mih5/p/564716
pro-SPC unconj: https://www.emdmillipore.com/US/en/product/Anti-Prosurfactant-Protein-C-proSP-C-Antibody,MM_NF-AB3786
MHCII unconj: https://www.biolegend.com/en-us/products/purified-anti-mouse-i-a-i-e-antibody-368
E-Cadherin unconj: https://www.abcam.com/e-cadherin-phospho-s838--s840-antibody-ep9132y-ab76319.html

Eukaryotic cell lines

Policy information about cell lines

Cell line source(s)	The B6 skin fibroblast cell line was generated in our laboratory, published in Sinnathamby et al., J Immunol, 2004. The T cell hybridoma cell lines were generated in our laboratory, published in Tewari et al., Nat Immunol, 2005, and Miller et al., Nat Med, 2015. The MDCK cell line was provided by S. Hensley (University of Pennsylvania), originally obtained from the National Institutes of Health. The LLC-MK2 cell line was obtained from C. Lopez (University of Pennsylvania), originally from ATCC (Cat # CCL-7).
Authentication	All of the cell lines appeared as expected morphologically by light microscopy. All cells were also phenotypically as expected - MDCK cells supported robust influenza virus replication, and LLC-MK2 cells supported robust Sendai virus replication. The B6 fibroblasts demonstrated absent MHCII expression by flow cytometry, as expected, and the T cell hybridomas responded to their cognate peptides.
Mycoplasma contamination	LLC-MK2 cells and B6 fibroblasts tested negative for mycoplasma contamination. MDCK cells and T hybridomas were not tested.
Commonly misidentified lines (See ICLAC register)	No commonly misidentified cell lines were used in the study.

Animals and other organisms

Policy information about studies involving animals; ARRIVE guidelines recommended for reporting animal research

Laboratory animals	Laboratory mice of the strains listed below were used for experiments (also listed in methods section). Mice were age and sex matched for all studies and 6-12 week old mice were used for all experiments, unless otherwise specified. Both male and female mice were used for ex vivo studies, but only female mice were used in all in vivo weight loss and survival experiments to facilitate co-housing between mice of different strains.<br><br>C57Bl/6 wild-type (B6), B6.129S2-H2dlAb1-Ea/J (MHCII-/-), B6;129S4-H2-DMatm1Luc/J (H2-DMa-/-), B6.129S7-Ifngtm1Ts/J (Ifng-/-), B6.129S(Cg)-Stat1tm1Dlv/J (Stat1-/-), C.129S2(B6)-Ciitatm1Ccum/J (Ciita-/-), B6.SJL-Ptprca Pepcb/BoyJ (CD45.1 B6), B6.129X1-H2-Ab1tm1Koni/J (H2-Ab1fl/fl), BALB/c wild-type, CB6F1/J (F1 [BALB/c x C57Bl/6] mice were originally purchased from the Jackson Laboratory. C57Bl/6 Cd74-/- mice were originally provided by Guo-Ping Shi (Harvard), C57Bl/6 Ciita pIV-/- mice were provided by S. Hugues (University of Geneva), and C57Bl/6 H2-Ob-/- mice were provided by L. Denzin (Rutgers). C57Bl/6 SPC-Cre-ERT2 mice were provided by G.S. Worthen, B6.129S7-Ifngr1tm1Agt/J (Ifngr1-/-), B6(Cg)-Ifnar1tm1.2Ees/J (Ifnar1-/-), and B6.Cg-Ifngr1tm1Agt Ifnar1tm1.2Ees/J (Ifnar1-/-Ifngr1-/-) mice were provided by E. Behrens, and germ-free mice were provided by M. Silverman (Children's Hospital of Philadelphia). C57Bl/6 B6.129P2(SJL)-Myd88tm1.1Defr/J (Myd88-/-) mice were provided by S. Shin, and B6.129S2(C)-Stat6tm1Gru/J (Stat6-/-) mice were provided by C. Hunter (University of Pennsylvania).
Wild animals	No wild animals were used.
Field-collected samples	No field collected samples were used.
Ethics oversight	All studies were approved by the Institutional Animal Care and Use Committee (IACUC) at the Children's Hospital of Philadelphia.

Note that full information on the approval of the study protocol must also be provided in the manuscript.

Human research participants

Policy information about studies involving human research participants

Population characteristics	In the general study protocol, alveolar type 2 cells are obtained from donor lungs from adult (18-70yo) subjects declared brain dead, whose lungs are without underlying pulmonary disease but are deemed not suitable for lung transplantation. The one subject included in this study was a 36 y.o. M with head trauma from a motor vehicle accident, who had no smoking history or evidence of active infection, with a P/F ratio of 346.
Recruitment	Donor lungs were obtained as part of recruitment in the Prospective Registry of Outcomes in Patients Electing Lung Transplantation Study, which recruits donors being assessed for lung transplantation. Lungs are procured via voluntary Gift of Life donation. With this recruitment method, there may be variables unaccounted for that are more likely to occur with willingness or ability to donate, such as geographic proximity to transplantation center, socio-economic status, race, sex, etc. However, these variables are unlikely to have influenced or biased the analyses of one healthy donor used in our study.
Ethics oversight	University of Pennsylvania Institutional Review Board

Note that full information on the approval of the study protocol must also be provided in the manuscript.

Flow Cytometry

Plots

Confirm that:

- [x] The axis labels state the marker and fluorochrome used (e.g. CD4-FITC).
- [x] The axis scales are clearly visible. Include numbers along axes only for bottom left plot of group (a 'group' is an analysis of identical markers).
- [x] All plots are contour plots with outliers or pseudocolor plots.
- [x] A numerical value for number of cells or percentage (with statistics) is provided.

Methodology

Sample preparation	See methods section for a detailed description of how single cell suspensions were prepared for flow cytometry from mouse lung, spleen, blood, and human distal lung.
Instrument	LSRII and LSRFortessa analyzers with FACSDiva software (BD)<br>Cytoflex S and Cytoflex LX analyzers with CytExpert software (Beckman Coulter)<br>FACSAria Fusion sorter with FACSDiva software (BD)<br>FACSJazz sorter with FACS Sortware software (BD)<br>MoFlo Astrios sorter with Summit software (Beckman Coulter)
Software	The specific software used to acquire data with each instrument is described directly above in the "Instrument" section. After acquisition, all data were analyzed using FlowJo 10 software (FlowJo LLC).

Cell population abundance	Post-sort or post-magnetic bead depletion purity checks were performed by flow cytometry for the following experiments:<br>DQ-Ova assay: >95% purity<br>Hybridoma assay: >90% purity<br>ELISpot assay: APCs >95% purity; T cell subsets 90% purity overall with <2% contaminating T cells from the other subset<br>Cathepsin D assay: AT2s ~90% purity, B cells ~80% purity<br>Cathepsin L assay: AT2s ~97% purity, B cells ~95% purity
Gating strategy	For all experiments, debris was gated out using a FSC vs SSC gate, and doublets were also excluded via FSC-A vs FSC-H, FSC-A vs FSC-W, or FSC-H vs FSC-W gates. For all analytical experiments, dead cells were also excluded by staining with a Live/Dead viability dye. For cell sorting experiments, a viability stain was not included in order to minimize sample processing time.<br><br>The gating strategies for each cell population studied are described in detail in the methods section based on the markers used to identify each cell type. Representative gating strategies are shown in Supplementary Figures 1, 2, 10, 11, 12, 13.

☒ Tick this box to confirm that a figure exemplifying the gating strategy is provided in the Supplementary Information.
